# Analysis of the three dimensional structure of the kidney glomerulus capillary network

**DOI:** 10.1038/s41598-020-77211-x

**Published:** 2020-11-23

**Authors:** Mark Terasaki, Jason Cory Brunson, Justin Sardi

**Affiliations:** 1 Department of Cell Biology, University of Connecticut Health Center, Farmington, CT 06030 USA; 2 Laboratory for Systems Medicine, University of Florida Health, Gainesville, FL 32610 USA

**Keywords:** Cell growth, Cellular imaging

## Abstract

The capillary network of the kidney glomerulus filters small molecules from the blood. The glomerular 3D structure should help to understand its function, but it is poorly characterized. We therefore devised a new approach in which an automated tape collecting microtome (ATUM) was used to collect 0.5 μm thick serial sections from fixed mouse kidneys. The sections were imaged by scanning electron microscopy at ~ 50 nm/pixel resolution. With this approach, 12 glomeruli were reconstructed at an x–y–z resolution ~ 10 × higher than that of paraffin sections. We found a previously undescribed no-cross zone between afferent and efferent branches on the vascular pole side; connections here would allow blood to exit without being adequately filtered. The capillary diameters throughout the glomerulus appeared to correspond with the amount of blood flow within them. The shortest path (minimum number of branches to travel from afferent to efferent arterioles) is relatively independent of glomerular size and is present primarily on the vascular pole size. This suggests that new branches and longer paths form on the urinary pole side. Network analysis indicates that the glomerular network does not form by repetitive longitudinal splitting of capillaries. Thus the 3D structure of the glomerular capillary network provides useful information with which to understand glomerular function. Other tissue structures in the body may benefit from this new three dimensional approach.

## Introduction

There are approximately 1 million nephrons in a human kidney (e.g.,^1^). Their collective function is to maintain the homeostatic concentration of ions and small molecules in the blood. The first part of the nephron is the glomerulus, a capillary “tuft” enclosed within Bowman’s capsule. The capillary network performs the initial filtration of water, ions and small molecules from the blood. In a healthy person, 20% of the cardiac output goes to the kidneys, of which 10–15% is filtered by the glomerulus (the glomerular filtration rate). The filtrate passes through the tubular system of the nephron, where needed materials are resorbed and ion concentrations adjusted, leaving toxic or uneeded substances which will be excreted. Glomeruli are directly damaged by auto-immune diseases or toxic chemicals and are secondarily damaged in diseases such as diabetes and hypertension. Severe damage leads to end-stage renal disease (ESRD), a leading cause of death.

A single input (afferent) arteriole feeds into the glomerulus and a single output (efferent) arteriole leads away from it. The afferent arteriole branches to form a complex network of capillaries that converges back to the efferent arteriole. It has been difficult to document the exact branching pattern of the capillaries. Paraffin sections, the standard histological method, are typically 4–5 μm thick or thicker. This is not thin enough to reconstruct the capillaries in three dimensions.

There have been several approaches to this problem. The glomerular network has been imaged by X-ray nanotomography^2^, but the exact branching pattern in the interior was not determined. The glomerulus was embedded in epon, cut in 1 μm thick serial sections and imaged by light microscopy with a high numerical aperture objective lens^3^. This was so challenging that only one glomerulus was analyzed. An intravital approach used an experimental hydronephrosis which destroys the tubules and makes the glomerulus visible by video microscopy^4^. The capillary branches on the upper surface of the glomerulus were deduced by the paths taken by red blood cells. However, the paths in the deeper half could not resolved and only half of one glomerulus was analyzed. Other studies have similarly reconstructed only one or have done partial reconstructions (see “Discussion”). Thus, the capillary network has not yet been adequately characterized in three dimensions.

Recently developed techniques for serial section electron microscopy offer new ways to investigate the three dimensional structure of biological structures. This is made possible by automated collection, as well as radical improvements in computer speed and software for alignment and three dimensional analysis. The ATUM tape collector method picks up sections on tape and the sections are imaged by a scanning electron microscope. Using this method, 2000 sections, each 30 nm thick, were cut and collected from mouse brain cortex^5^. A 50 µm × 50 µm field of view was imaged at 3 nm per pixel resolution (16,000 × 16,000 pixel images). We have adapted the ATUM method^5^ for the glomerulus, and have reconstructed 12 glomeruli. Network analysis (classically known as graph theory) can be very useful for analyzing glomerular connectivity^6,7^, and we used some of these tools to analyze glomerular properties.

## Results

### 500 nm sections

The ATUM tape collector method picks up sections on tape and the sections are imaged by a scanning electron microscope. Using this method, 2000 sections, each 30 nm thick, were cut and collected from mouse brain cortex^5^. A 50 µm × 50 µm field of view was imaged at 3 nm per pixel resolution (16,000 × 16,000 pixel images).

A similar approach could be used on the mouse glomerulus, which is approximately 70 μm in diameter. However, even a single glomerulus would be a major undertaking due to the difficulty of collecting several thousand sections and lengthy imaging time. We realized that cutting thicker sections and imaging at lower resolution would still provide enough information to reconstruct the glomerular capillary network, and would allow us to image multiple glomeruli to compare their structures.

For imaging by transmission electron microscopy, sections must be thin enough so that electrons can pass through, typically 60–70 nm. With scanning EM, the image signal is formed from electrons that are scattered back from the superficial layers of the section surface. Thus, the surface of sections of arbitrary thickness is easily imaged. A 70 µm diameter glomerulus cut into 0.5 µm thick sections requires only 140 sections. Although high resolution imaging (e.g. 5 nm per pixel) is somewhat compromised with thick sections, a moderate image resolution of 50–100 nm per pixel (which is considered to be “super-resolution” by light microscope standards) is adequate to resolve cell boundaries and many tissue features.

**Figure 1:**
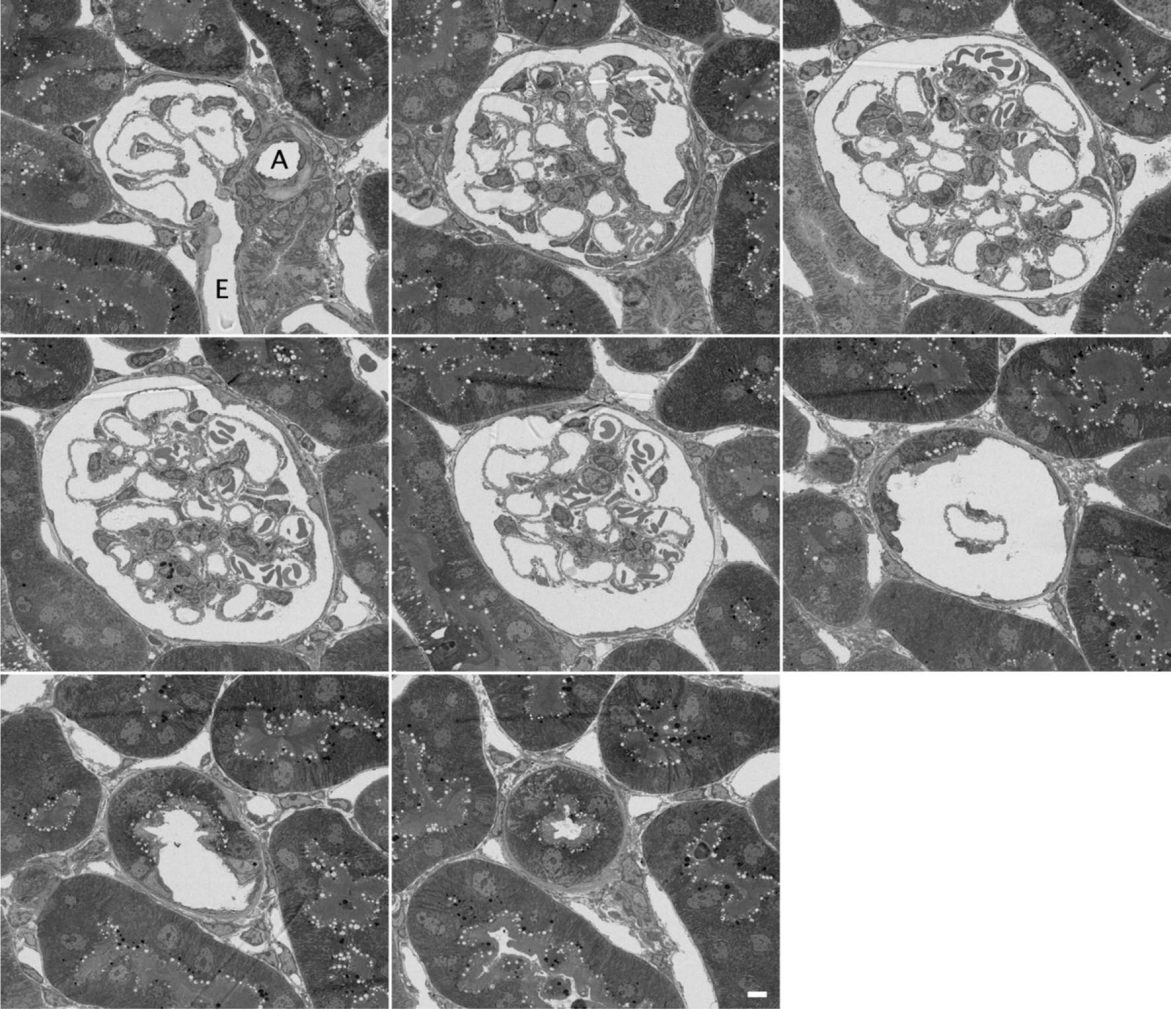
Montage of serial section electron micrographs. The sections were 500 nm thick, and were imaged at 66 nm per pixel. The section numbers were #159, 137, 106, 80, 55, 30, 16 and 6 out of 238 total sections. “A” marks the afferent arteriole, and “E” denotes the efferent arteriole. The urinary pole region is shown in the last two panels. These came from glomerulus #6 of Fig. 2. Scale bar 5 µm.

**Figure 2:**
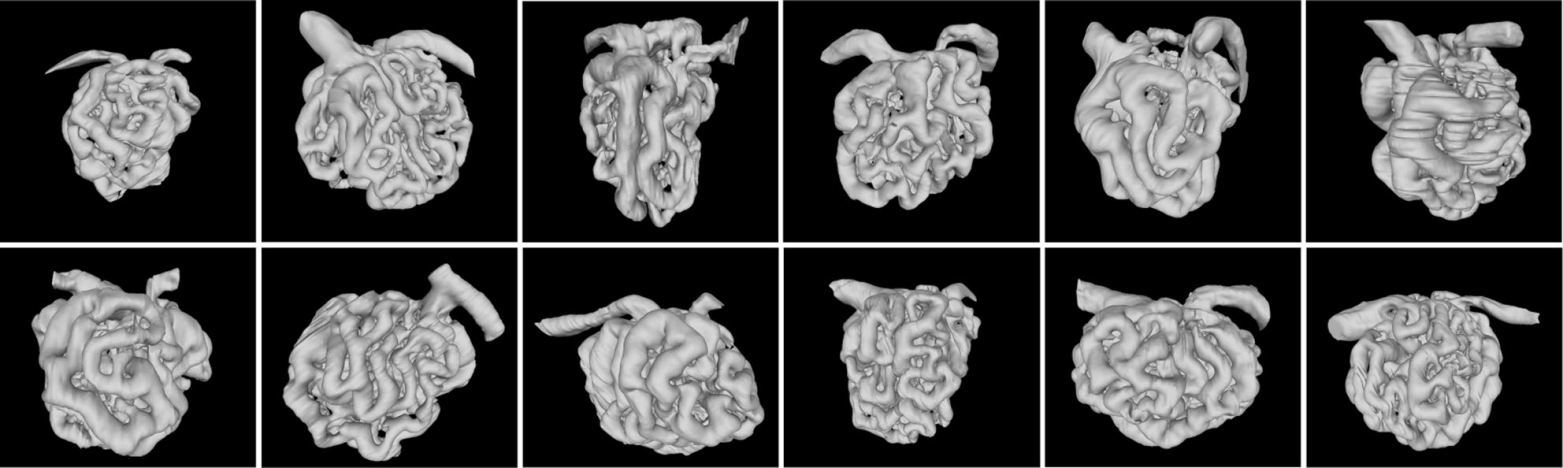
Montage of reconstructions of 12 glomeruli. Serial section data was segmented then 3D models were generated. They are shown from smallest (fewest nodes) to largest. Each glomerulus is oriented such that the vascular pole is up, the urinary pole is down, the afferent arteriole is on the left and the efferent arteriole is on the right.

Serial section images of the mouse glomerulus show the capillary network clearly (Fig. 1). The arterioles at the vascular pole as well as the beginning of the proximal tubule at the urinary pole are easily identified. The afferent arteriole entering the glomerulus can be identified because it branches from a large artery, whereas the efferent arteriole dives down into the medulla of the cortex. We collected serial sections of 12 glomeruli from 4 different mice. For each, the capillary lumen was segmented, reconstructed and rendered (Fig. 2); in the figure, the glomeruli are all oriented with the vascular pole up, urinary pole down, afferent arteriole to the left and efferent arteriole to the right.

### Features of glomerular capillaries

**Figure 3:**
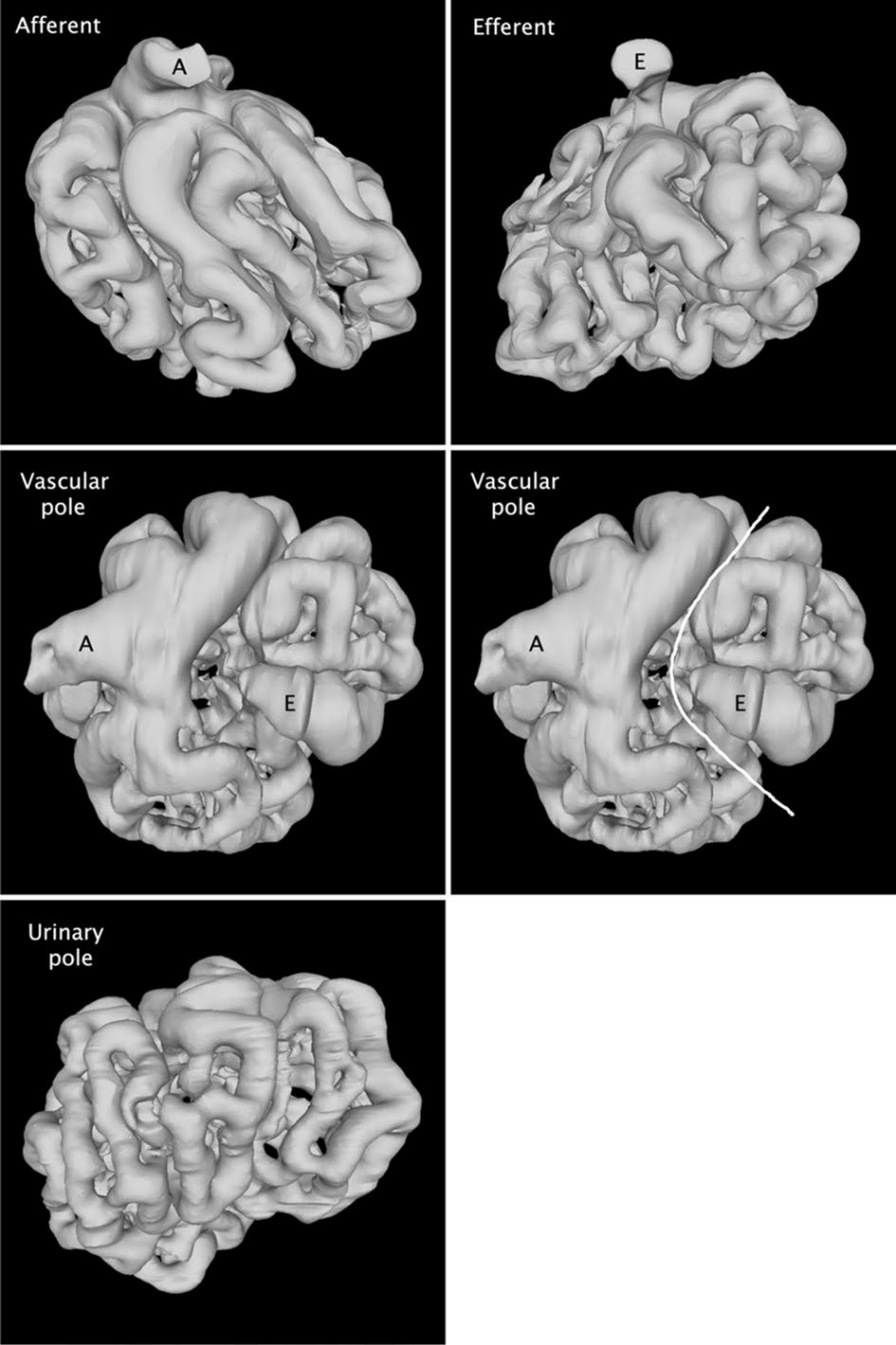
Views from several sides of a reconstructed glomerulus (far left glomerulus in the second row of Fig. 2). The afferent and efferent arterioles are indicated by the letters “A” and “E” respectively. Top row: the afferent arteriole branches (left panel) are noticeably wider than the efferent arteriole branches (right panel). Middle row: view from the vascular pole side, showing the branches from the afferent arteriole and efferent arteriole. The wider afferent arteriole branches are also evident from this view. The white line on the right panel indicates the “no-cross zone” or cleft which separates the afferent branches and efferent branches. If a connection existed here, it would constitute a short cut path which would allow blood to pass through without being filtered. Bottom row: view from the urinary pole shows that the capillary segments are narrower than those on the vascular pole side, which corresponds to the expected lower blood flow rate in these segments.

It is commonly stated that the afferent arteriole is wider than the efferent arteriole, corresponding to the loss of volume due to the blood filtration by the glomerulus. In the reconstructions, the afferent and efferent cross sectional areas were 444 ± 266 µm^2^ vs 150 ± 60 µm^2^, and the ratio of these areas was 2.96 ± 1.08 (N = 12). By visual inspection, the glomerular branches of the two arterioles are also correspondingly different in width (Fig. 3, top row).

When the reconstructed glomeruli are viewed from the vascular pole side (i.e., the side with the two arterioles), there is invariably a gap which is roughly perpendicular to the plane containing the afferent and efferent arterioles (Fig. 3, middle row). The existence of this cleft provides a rationale for dividing the glomerulus into an afferent side and an efferent side, and has interesting consequences. First of all, the existence of a “no-cross zone” is consistent with glomerular function, because a connection in this region would provide a path for blood to exit without being filtered.

Secondly, as the afferent side capillaries bifurcate in their path to the urinary pole, each new branch must be carrying a lower amount of blood. Conversely, on the efferent side, the converging capillary segments must carry an increasing amount of blood. It follows then, that the capillary segments at the urinary pole must have the lowest blood flow in the glomerulus. These are noticeably narrow (Fig. 3, bottom row). Just as the afferent and efferent arteriole diameters vary with the amount of blood flowing through them, the glomerular capillary diameters appear to vary with the blood flow.

### Application of network theory

**Figure 4:**
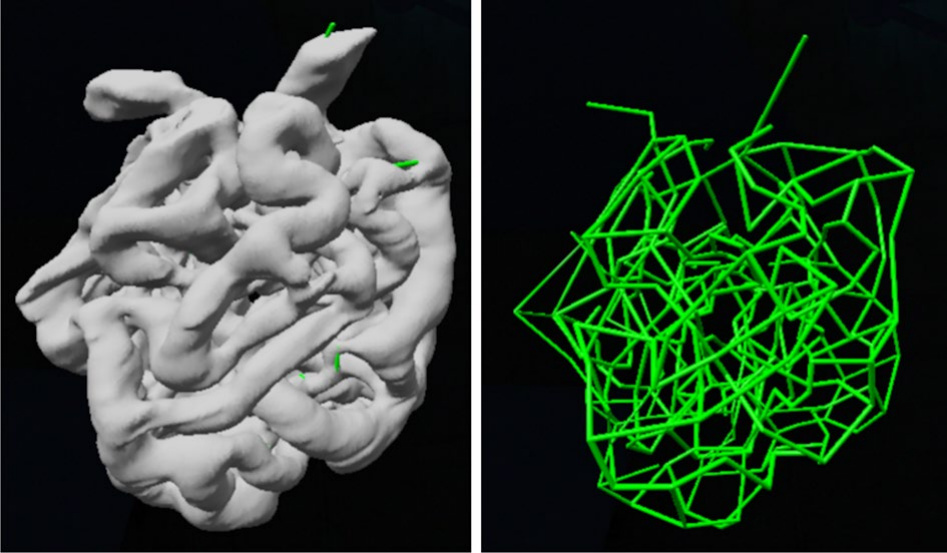
Three dimensional network diagram constructed from a glomerular reconstruction. The nodes and connections were identified and constructed in a 3D virtual reality system.

Wahl et al.^6^ have pointed out that network theory (also called graph theory) is well suited for investigating properties of glomeruli. The first step in this kind of analysis is to document the nodes (branch points) and edges (capillary segments). A virtual reality system was used to inspect reconstructions (.obj files) in order to document the nodes and how they are connected (Fig. 4) as well as to create an “adjacency list” for network analysis.

We point out that the above reasoning about branching and flow can be recapitulated by calculating the electric current in a network of resistors having the same branching pattern as a glomerulus. This is a standard problem in electric circuit analysis and is solvable by elementary matrix methods^8^. As expected, the electrical current is lowest for the paths mid-way between the input and output.

**Figure 5:**
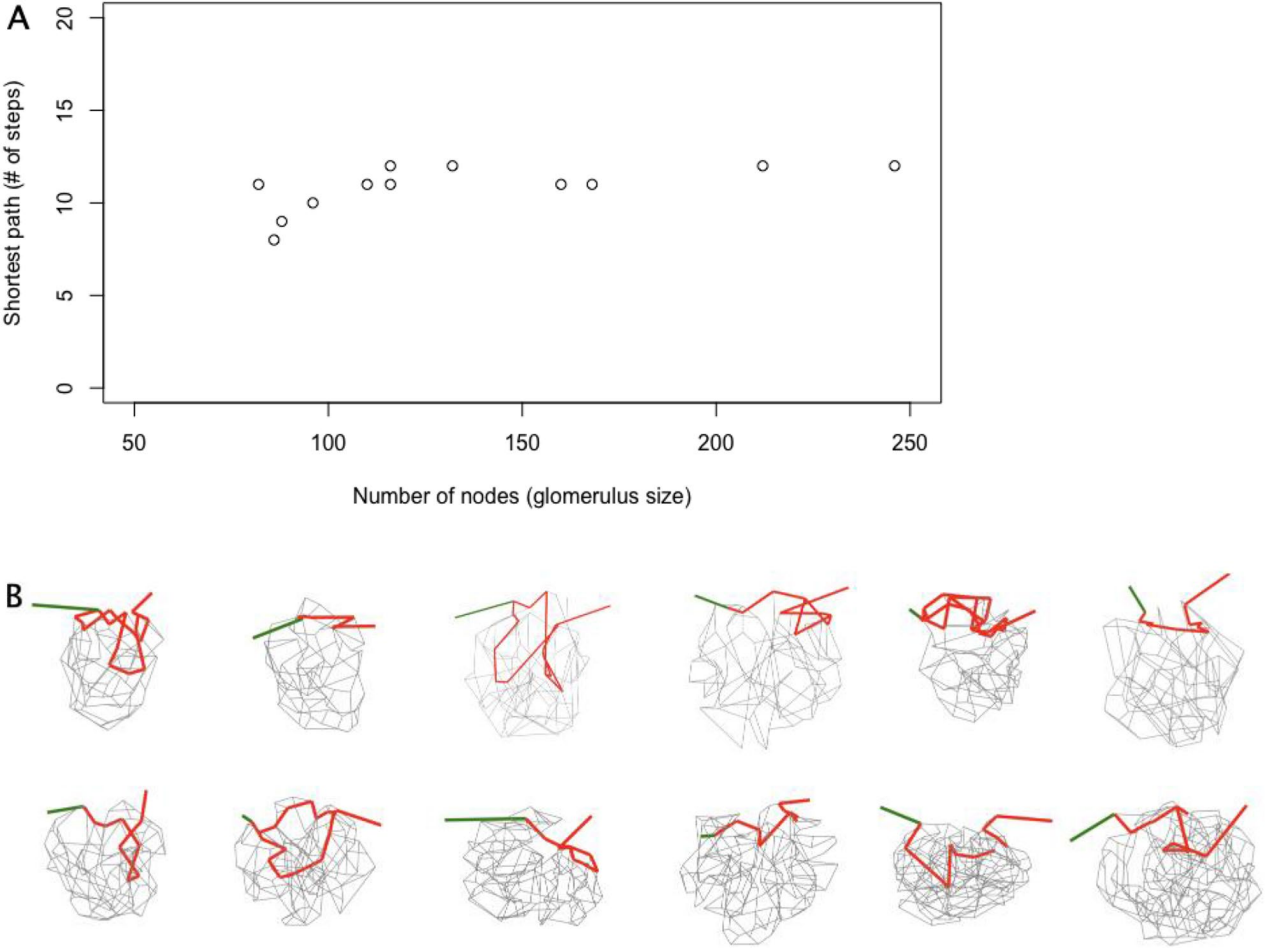
Shortest path versus glomerulus size. (**A**) Graph of the number of steps in the shortest path versus the number of nodes (branch points) in the glomerulus. (**B**) The glomeruli are shown in the same order as in Fig. 2, and oriented similarly with the afferent arteriole on the left colored green. The shortest path(s) (in several instances, there are two paths of the same length) from afferent to efferent arteriole is shown in red, and the other connections within the glomerulus are shown in gray. The shortest path is located on the vascular pole side of the glomerulus. These observations suggest that the shortest path is established at some point during glomerular development, after which more branches are added on the vascular pole side which create paths of longer lengths.

An important network parameter is the shortest path, that is, the minimum number of nodes traversed by a path from the afferent arteriole entry to the efferent arteriole exit. We determined this using standard network methods^8^. The number of steps in the shortest path ranged from 8 to 12. When plotted against the number of nodes, the shortest path is largely independent of the number of nodes (Fig. 5A). Wahl et al.^7^ made a similar observation from their analysis of 6 glomeruli from different studies.

When the shortest path(s) (sometimes there are two paths of the same length) is plotted, it is present in on the vascular pole side of all of the glomeruli (Fig. 5B), avoiding the no-cross zone. This and the relatively constant number of steps in the shortest path suggests the shortest path is established at some point of glomerulus development, and that after this time, branches are added on the urinary pole, creating paths of longer lengths.

**Figure 6:**
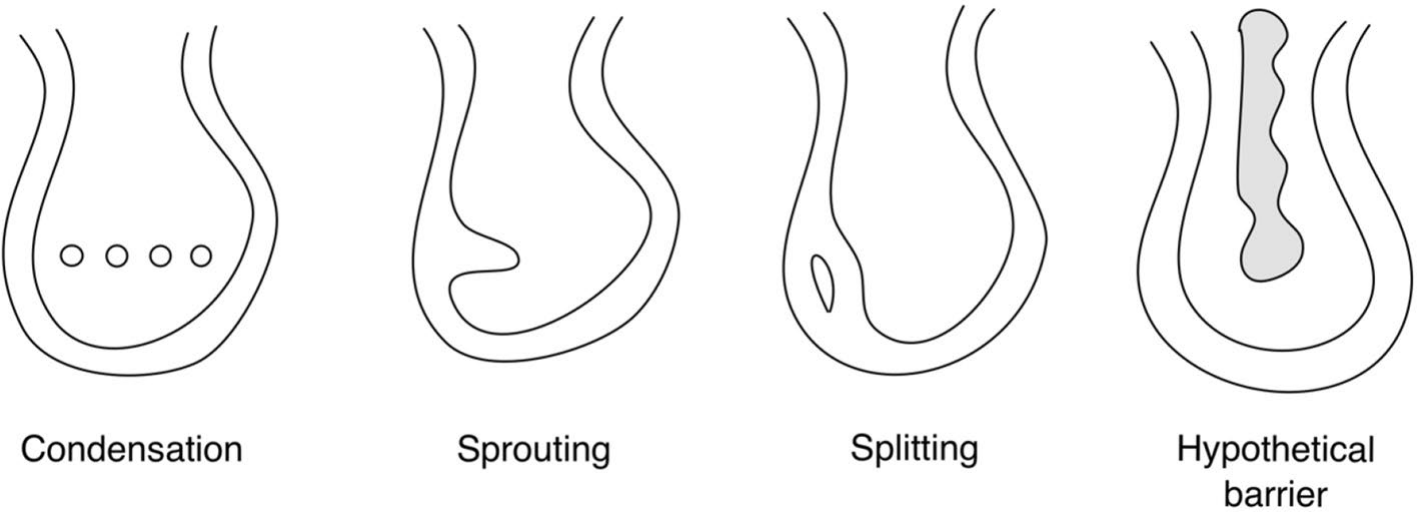
Three different ways to form capillary branches. From left to right: condensation of endothelial cell precursors, also called vasculogenesis; sprouting from pre-existing capillary; Widening of a capillary region following by longitudinal splitting. Far right: hypothetical barrier that could prevent formation of capillary branches which would short circuit the glomerulus.

Lastly, network theory can be used to evaluate a hypothesis concerning glomerular development. The capillary nework is thought to begin as a single loop which then branches^9^. There are three ways to add branches^10,11^: coalescence of mesenchyme cells (vasculogenesis), sprouting (angiogenesis), and splitting (intussusception) (Fig. 6). It occurred to us this that this could be a consequence of longitudinal splitting of capillaries to form branches (e.g.^12^).

We used network theory to test this hypothesis. Repeated splitting also produces a “flat” network, i.e., one that can be drawn on paper without any crossing lines. An algorithm that detects planar graphs using the Kuratowski theorem (e.g.,^13^) shows that all 12 glomeruli are not flat. A similar conclusion was reached by Wahl et al.^6^ who analyzed six glomeruli from several different publications. We conclude that repeated longitudinal capillary splitting is not the sole mechanism for preventing short cuts nor for constructing the glomerulus network, though it is certainly possible that splitting may occur in parallel with sprouting or condensation. We present another hypothesis for preventing short cuts that involves a physical barrier inside the initial glomerular loop (see “Discussion”).

## Discussion

Why is the glomerulus a branched network, not a single long convoluted capillary? Two explanations, not necessarily exclusive, are redundancy and filtration efficiency. A branching network provides redundant pathways that could circumvent damage or an occlusion. A branching network also could filter more efficiently by exposing the blood to a greater amount of surface area and/or increasing the volume that can be filtered per unit time.

Since the glomerulus is in fact a branched network, does the exact type of branching pattern have significance? The flow biophysics can be very complex, added to the fact that the pressure in the glomerular capillaries is higher than in the peripheral capillaries^14^.

Questions like these could be better addressed if the connectivity of the glomerular capillaries were known. A number of studies have therefore been devoted to obtaining reconstructions^3,4,15–17^. Most recently, careful partial reconstructions of human glomeruli have been made^18^. Advances in computer capabilities facilitate making reconstructions, and network theory is well suited for analyzing the connectivity^6^.

We have used a new approach involving serial section electron microscopy to fully reconstruct 12 mouse glomeruli. Visual inspection of the reconstructions shows that there is a “no cross zone” which is roughly perpendicular to the afferent/efferent arteriole axis. This is an important feature of the glomerulus because connections in this region could provide a short cut for blood to pass through without filtration. Due to the existence of this previously undocumented cleft, it now makes sense to refer to the afferent and efferent side of the glomerulus.

We noticed that the arteriole and capillary diameters correspond with the expected blood flow rate within them. It is already accepted that the afferent arteriole is wider than the efferent arteriole due to filtration loss of fluid. From the reconstructions, the capillary segments leading from the afferent arteriole are likewise wider than the capillary segments feeding into the efferent arteriole. Furthermore, since flow is reduced when a capillary splits (afferent side) or increased when capillaries combine (efferent side), the flow should be lowest in the capillary segments mid way between the afferent and efferent arterioles, i.e., in the urinary pole region. Correspondingly, the segments in this region are the narrowest in the glomerulus.

Much work indicates that blood vessels adapt to characteristics of fluid flow^19^. Generally, laminar (smooth) flow is thought to be optimized while disrupted/turbulent flow minimized. In the glomerulus, the network topology could determine, to first approximation, the flow rate in the different branches, and the capillaries could adjust their diameter or other properties in order to promote laminar flow.

Blood flow in the glomerulus and its relation to filtration is very complex, involving such factors as the pulsatile changes in pressure, turbulence at branch points, and possible transient reversals of flow direction^4,20^. Establishing the connectivity of the capillaries should help to make progress in understanding these factors.

Wahl et al.^6^ pointed out the usefulness of network/graph theory in studies of the glomerulus. We used some of these methods as an aid to analyzing the the glomerulus connectivity and to test a hypothesis of its development. The minimum number of nodes that must be traversed from afferent to efferent arteriole (the shortest path) was determined for the 12 glomerular networks using standard network theory analysis. Strikingly, this seems relatively independent of glomerular size (Fig. 5A). A similar observation was made earlier based on a smaller number of glomeruli from several experimental studies^7^. This suggests that the glomerulus grows to a certain size, after which, additional branches do not alter the shortest path (i.e., do not make it longer) but instead only make longer alternative paths. When we plotted the shortest paths, they were found to exist almost entirely in the vascular pole side of the glomerulus (Fig. 5B). This suggests that paths/later branches are added at the urinary pole.

We also used a well-known theorem of network theory to evaluate an idea about glomerulus development. As described in “Results”***, short cuts between the afferent and efferent arterioles would never form if the glomerular network arose from repeated capillary splitting. One abstract feature of the resultant network is that it is “planar”, i.e., can be drawn on a piece of paper without any overlaps. Network analysis shows that the actual glomerular network is not, which eliminates this hypothesis.

We suggest another mechanism. The initial glomerular structure forms an S shape^9^. Blood vessel endothelial cells move into the empty region of the lower S and become associated with the layer that will become the podocytes. In that region, a capillary loop forms. Subsequent branching, which depends on PDGF^21^, forms the glomerulus. A structure positioned perpendicularly in the interior loop could form a physical barrier to blood vessel growth (diagram on the far right of Fig. 6). For instance, the structure could be precursors to the mesangium. This would allow branching on the afferent and efferent sides but not across the barrier, and would prevent formation of short cuts.

### Three dimensional histology

The glomerulus is a classic subject of histology, the field of biomedical science that studies the organization of cells in tissues. The histological approach was established by Virchow^22^, and remains an essential part of pathology. Histological considerations are important for regenerative biology which depends crucially on the organization of cells in stem cell niches.

The primary methodology is stained paraffin section, which gives very good views of cell organization. It is surprising though, that histology textbooks illustrate the three dimensional organization of tissues by the use of drawings or diagrams rather than reconstructions. Students are trained to “imagine” the three dimensional structure from single paraffin sections.

This situation is due to the fact that paraffin sections are typically ~ 5 µm thick. Because cells are approximately this size, paraffin sections are usually too thick to allow 3D reconstructions with cellular resolution. Additionally, the highest resolution images of paraffin sections are obtained with an oil immersion lens, with an xy resolution of ~ 0.5 µm or slightly less. By using the ATUM method to collect 500 nm thick sections, and imaging them with a scanning electron microscope at 50 nm per pixel, the images have 10 times the resolution of a paraffin serial section data in x, y and z dimensions. Thus many of the classic subjects of histology may benefit from three dimensional studies using this approach.

The serial section approach we used is one of several that were developed by neurobiologists to determine synaptic connectivity^5,23,24^. These methods involved several technical innovations and took advantage of tremendous advances in imaging technology and computer processing power. When used at a lower resolution, the new serial section methods provide an efficient and effective means for investigating biological organization at the cellular level.

## Methods

Three month old C57Bl/6J male mice under anesthesia were fixed by gravity fed cardiac perfusion of Karnovsky fixative (2.5% glutaraldehyde, 1% paraformaldehyde in 100 mM sodium cacodylate, pH 7.4) All procedures were approved by the animal care committee at UConn Health. All methods and procedures were performed in accordance with the relevant guidelines and regulations.

After several hours in fixative, kidney pieces were treated with 1% osmium, 0.8% potassium ferricyanide in 100 mM sodium cacodylate for 1 h, in 1% uranyl acetate in water overnight, stained with lead aspartate^25^ for 30 min at 60 °C, then dehydrated, infiltrated with epon resin and polymerized. The twelve reconstructed glomeruli were from four mice.

An EM UC-7 ultramicrotome (Leica, Buffalo Grove, IL) with a 6 mm wide histo diamond knife (Diatome, Hatfield, PA) was used cut 500 nm thick sections. Serial sections were collected with an ATUM tape collector (custom built by Ken Hayworth) on kapton tape. The tape was mounted on a silicon wafer (University Wafers, South Boston, MA) then carbon coated (Denton 502B, Moorestown, NJ). 200–250 sections collected.

A Verios (FEI, Hillsboro, OR) or Sigma (Zeiss, Thornwood, NY) field emission scanning electron microscope was used in backscatter mode to collect images. The ATUM approach keeps all the sections, so we searched in the cortex to find glomerulus completely included within series. This is less feasible with block face.

For mapping the sections and image collection with the Verios, the MATLAB program SEM Navigator (kindly provided by Daniel Berger, Harvard University) was used. On the Zeiss, custom Python (http:www.python.org) and Image J (https://imagj.nih.gov) scripts were used to map the sections and Atlas 4 (Fibics Inc., Ottawa, Ontario, Canada) was used to collect the images.

The images were aligned using Linear Stack Alignment with SIFT algorithm of FIJI Image J (https://fiji.sc/). The TrakEM2 module of FIJI was used for segmenting the images. MeshLab (https://meshlab.net) was used for rendering.

For network analysis, it is necessary to identify the nodes (intersection points) and how they are connected by edges (capillary branches). It was unexpectedly difficult to do this from the original serial section images. Essentially, the network is viewed from only one angle, and this makes it easy to mis-identify branch points or lose track of the path of the branches. Keeping track of nodes and connections within the data set was also problematic. We therefore worked with the reconstructions (.obj files) using a software that used virtual reality optics^26^ (SyGlass, Morgantown, WV). This allowed us to rotate, slice through, and place nodes and connections within the reconstruction. The software exported the node positions and connections for network analysis. The analysis was done in R (https://www.R-project.org) using the igraph (https://igraph.org) and RBGL (https://www.bioconductor.org) packages. The Kirchoff analysis was done in Matlab (https://www.mathworks.com). The display (Fig. 5A) was made using D3/javascript (https://d3js.org).

The length (number of nodes) of the shortest path was determined by multiplying the adjacency matrix by itself until the afferent and efferent arterioles were connected. Then the adjacency list was used to identify the nodes in the shortest path by tracing backwards from the efferent arteriole.
